# Comparative Neuroprotective Potential of Nanoformulated and Free Resveratrol Against Cuprizone-Induced Demyelination in Rats

**DOI:** 10.1007/s12035-024-04415-x

**Published:** 2024-08-17

**Authors:** Sara A. M. El-Sayed, Ghadha Ibrahim Fouad, Maha Z. Rizk, Hanan H. Beherei, Mostafa Mabrouk

**Affiliations:** 1 Refractories, Ceramics and Building Materials Department, National Research Centre, 33 El Bohouth St., Dokki, PO Box 12622, Cairo, Egypt; 2 Department of Therapeutic Chemistry, Pharmaceutical and Drug Industries Research Institute, National Research Centre, 33 El Bohouth St., Dokki, PO Box 12622, Cairo, Egypt

**Keywords:** Multiple sclerosis, Demyelination, Resveratrol, Magnetite nanoparticles, Cuprizone

## Abstract

Demyelination is a frequent yet crippling neurological disease associated with multiple sclerosis (MS). The cuprizone (CZ) model, which causes demyelination through oxidative stress and neuroinflammation, is a popular tool used by researchers to examine this process. The polyphenol resveratrol (RESV) has become a promising neuroprotective agent in seeking for efficient therapies. In a rat model given CZ, we created and examined iron oxide nanoparticles (IONPs) loaded with RESV (IONP-RESV) to see how effective they were as a therapeutic agent against free RESV. According to molecular mechanisms, exposure to CZ resulted in a marked downregulation of myelin proteolipid protein (PLP) expression and an overexpression of the inflammatory markers tumor necrosis factor-α (TNF-α) and S100β, which are indicators of demyelination and neuroinflammation. It is remarkable that these CZ-induced alterations could be reversed by therapy with either RESV or IONP-RESV. Interestingly, IONP-RESV showed even stronger anti-inflammatory activity, as shown by a more noticeable downregulation of TNF-α and S100β expression. These results were confirmed by histopathological examination of the cerebral cortices. Our findings support the better neuroprotective benefits of RESV-loaded IONPs over free RESV in reducing demyelination and neuroinflammation brought on by CZ. Owing to their pro-remyelinating, anti-inflammatory, and antioxidant properties, RESV-loaded IONPs show promise as a neurotherapeutic intervention in the future for neurological diseases such as multiple sclerosis.

**Graphical Abstract:**

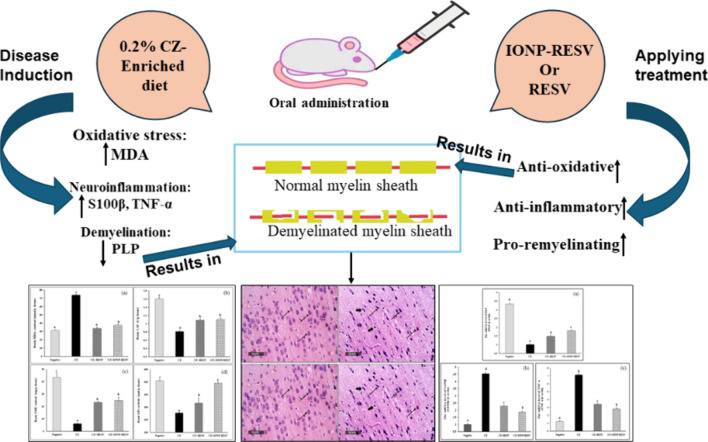

## Introduction

Copper (Cu), a cofactor of several “cuproenzymes,” exerts an important role in different cellular processes; disruption of Cu concentrations in the nervous system causes severe neurodegeneration [1, 2]. Cuprizone (CZ) induces neurotoxicity and demyelination in different brain regions; in addition, CZ disrupts the mitochondrial respiratory chain (complex IV), enhances the overproduction of reactive oxygen species (ROS) in the neural tissue, and subsequently mediates neuroinflammatory responses [3–5]. Multiple sclerosis (MS) is a neurodegenerative disorder with multifactorial pathological mechanisms, including myelin sheath loss “demyelination” and neuroinflammation [6].

Orally administered CZ is capable of provoking oligodendroglial apoptosis, glial cell activation, and demyelination via mitochondrial dysfunction [7]. By adjusting the CZ doses and the intoxication time frame, one can induce CZ-associated neurotoxicity in both murine and rodent models. Additionally, removing CZ from the diet triggers spontaneous remyelination in rat models, which allows for the evaluation of various factors on myelin restoration [8]. The well-known protocol of CZ intoxication (i.e., mixed in powdered chow) is the ideal model to achieve reproducible neurotoxicity [9]. However, the amount of CZ consumed “CZ-intake” by the animals might vary due to the loss of activity of CZ and its degradation by environmental factors, leading to a difference in the demyelination degree of each animal [10]. Resveratrol (RESV) can treat CZ-triggered demyelination and neuroinflammation [11–13].

Resveratrol [3,5,4′-trihydroxystilbene] is a polyphenolic compound that has two configurations: cis-RESV and trans-RESV. It exists as a monomer or as an oligomer of 2–4 monomers and is naturally present in grapes and berries. Researchers have found trans-RESV, a non-toxic stereoisomer, to exhibit a multitude of health-promoting benefits, including anti-oxidative and anti-inflammatory potentials [14–16]. Additionally, RESV demonstrated neuroprotective activities against several neurological disorders [11–13]. A recent trial (NCT01504854) has reported RESV to exert neurotherapeutic, anti-inflammatory, and immunomodulatory activities in patients with Alzheimer’s disease [17].

Nevertheless, the administration of RESV in its “free form” has limited in vivo bioavailability and efficacy. RESV is poorly soluble in water and belongs to class II of the Biopharmaceutics Classification System (BCS) [18]. In addition, RESV is photoinstable and is prone to auto-oxidation and the subsequent generation of complex semiquinones, quinines, and toxic phenantrenoids [19]. Biologically, the bioavailability of RESV relies on its administration route; orally administered RESV is subjected to enterohepatic circulation, glucuronidation, and sulfation, which reduces the quantity of free RESV reaching the target tissues and limits its bioavailability in both rats and humans [20, 21]. Therefore, it was suggested that nanoformulation of RESV could enhance its bioavailability and efficacy; a recent study by [22] demonstrated that “nanoformulated” RESV was 800 and 3000 times more potent than “free” RESV.

Nanoformulation of therapeutic agents, such as RESV, is very interesting for treating neurological disorders because many therapeutic molecules cannot get into the brain [23]. Over 98% of small molecules and almost all the large molecules cannot cross the blood–brain barrier (BBB) [24]. Additionally, the invasive methods are expensive and are associated with side effects such as brain infections [25]. Therefore, nanotechnology-based delivery systems are considered alternative therapeutic approaches for brain disorders, enabling the combination of targeting, drug delivery, and drug release. Nanocarriers should be safe, able to load the bioactive molecules, and able to cross-link with receptors present at the BBB [26]. Several studies have developed effective nanosystems for the delivery of RESV across the BBB [27–29].

Iron oxide nanoparticles (IONPs) are the most commonly used inorganic nanoparticles in biomedical applications, primarily for drug delivery. IONPs with diameters between 1 and 100 nm are commonly employed in biological applications [30, 31] and are mainly in the magnetite (Fe_3_O_4_) form and its oxidized derivative maghemite (γ-Fe_2_O_3_) [32]. The challenge of drug delivery across the BBB is the size of membrane pores and drug size. However, most drugs that target the central nervous system (CNS) have limits of penetration because of their size. So, the most challenging target is the preparation of nanoparticles and loading the drug on these nanoparticles that have the ability to cross BBB. Biomedical applications use a variety of nanoparticles, including polymers and inorganic materials, due to their flexibility in surface, size, and shape modification [33]. IONPs have many advantages that promote their use in biomedical applications. This is due to their small size, high surface area, biocompatibility, and magnetic properties [34]. This magnetic characteristic of IONPs enhances the permeability of drug delivery across the neural cells [35, 36]. This study aimed to compare the neuroprotective potential of either free RESV or IONPs-RESV against CZ-induced demyelination and neuroinflammation in rats.

## Materials and Methods

### Materials

CZ (CAS No.: 370–81-0; ≥ 95%), RESV (CAS No.: 501–36-0; ≥ 99%), phosphate saline buffer (PBS, pH 7.4), and tween 80 were bought from Sigma, St. Louis, USA. Diagnostic kits were procured from Biodiagnostic, Egypt. Anhydrous iron chloride (FeCl_3_) (97%, MWt = 162.20 g/mol) was purchased from Sigma-Aldrich (Germany). Ethylene glycol ((CH_2_OH)_2_) (99%, MWt = 62.07 g/mol) was purchased from Alpha (India). Hydrated hydrazine (N_2_H_4_.H_2_O) (99%, MWt = 50.06 g/mol) was purchased from Advent (India). Absolute ethanol (C_2_H_5_O) (99.8%, MWt = 46.07 g/mol) was purchased from Piochem (Egypt).

### Methods

The preparation of magnetite IONPs was made as in our previous research [37]. Briefly, using a magnetic stirrer, anhydrous iron chloride was dissolved in ethylene glycol to make a light-yellow solution. After dissolving, we add a few milliliters of hydrated hydrazine, which turns the solution into a brown solution. The solution was then converted to a yellowish-brown suspension after 1 h of continued stirring. The suspension dried at 150 °C for 2 days using a Teflon-lined stainless-steel autoclave. Then, the magnetite was obtained using the centrifuge at certain conditions (7000 rpm at 20 °C for 15 min). The black precipitate was washed with distilled water and absolute ethanol several times, and then dried at 37 °C for 2 days.

### FTIR Analysis

IONPs and IONPs-RESV were analyzed using Fourier transformer infrared (FTIR) spectrophotometer to identify the chemical functional groups. The prepared materials were mixed with pure potassium bromide (KBr) powder to form pellets that were then analyzed by the FTIR machine (model FT/IR-6100 type A). The spectra were measured at a wave number range of 400–4000 cm^−1^.

### TEM Analysis

The morphology and particle size of the IONPs and IONPs-RESV were identified by using transmission electron microscopy (TEM) (JEOL, Japan, JEM-2100, Electron Microscope, TEM-HR). Five microliters of the dispersed IONPs and IONPs-RESV using ethanol was dropped on a copper grid, and after grid drying, TEM images were recorded.

### Particle Size Distribution and Zeta Potential

The light scattering method using the Zetasizer Nano ZS instrument (Malvern Instruments, UK, accessorized with a 633 nm laser) was used to identify the size distribution and the zeta potential of the IONPs-RESV. Malvern Instrument’s dispersion technology software (version 7.13) was used for data analysis and zeta potential. The position of the zeta cells containing the dispersed IONPs-RESV using deionized water was adjusted at 5.50 mm and 2.00 mm for measuring the size distribution and the zeta potential, respectively, at 25 °C.

### X-Ray Photoelectron Spectroscopy (XPS)

X-ray photoelectron spectroscopy (XPS) measurements were collected with a SPECS spectrometer instrument (XR-50 source with monochromator—Focus 600) using an Al Kα monochromatic radiation (EKα (Al) = 1486.7 eV) and a hemispherical analyzer. IONPs-RESV was placed on a sample holder with carbon tape and double-sided adhesive. The applied pressure is 1109 mbar (for the ultrahigh vacuum (UHV)). Data was analyzed with Casa XPS processing and ULVAC-PHI MultiPak software (ver. 9.0.1, Osaka, Japan) for quantitative analysis. XPS spectra were obtained (PHOIBOS 150 MCD NAP).

### *In Vitro* Drug Release

The in vitro release of RESV from IONPs using dialysis bags was performed three times in PBS. Briefly, plastic containers filled with 50 ml of PBS contained resveratrol-loaded IONPs dialysis bags and were kept at 37 °C. Withdrawal of 3 ml from these containers was performed after certain time intervals (2, 4, 6, 24, 72, 168, 336, and 672 h) and replaced by fresh PBS to evaluate the RESV release. The fluorescence intensity of RESV has been investigated using a Hitachi Fluorescence Spectrophotometer (F-7100, Japan) at an excitation wavelength of 356 nm. The fluorescence spectrum was recorded in the range of 365 to 700 nm with increment 0.2 nm, a PMT voltage of 500 V, and a scan speed of 1200 nm/min. In addition, the drug concentration was determined at the emission wavelength of 383 nm [38]. Finally, the cumulative release of RESV in the PBS solution was calculated using Eq. (1).

1 $$\%\;Cumulative\;release\;=\;\frac{W_R}{W_T}\times\;100$$

Where *W*_R_ is the total amount of RESV that has been released in the medium, including being sampled every time, and *W*_T_ is the total amount of RESV encapsulated in IONPs-RESV.

### Animals and Grouping

Forty male Wistar rats, 6 weeks old (100 ± 20 g), were acquired from the Animal House of the National Research Center (NRC), Egypt. Rats were acclimatized for 2 weeks, were allowed to have access to food and water, and were kept in standard conditions of temperature (23–25 °C) and humidity (50–60%) with regular 12 h light/12 h dark cycle. The animal protocol followed the National Research Council’s Guide for the Care and Use of Laboratory Animals (NIH Publications No. 8023, revised 1978), and the experiment was approved by the Ethical Committee of the NRC, Egypt (Approval No. 19–313). Only a 0.2% CZ-enriched diet was administrated to rats “CZ-neurotoxicated rats” for 5 weeks; CZ-diet was prepared by mixing 0.2% of CZ into the ground chow for 5 weeks, according to Omotoso et al. [39]. Chow was prepared fresh daily, and cages were changed every other day.

### Experimental Design

**Fig. 1:**
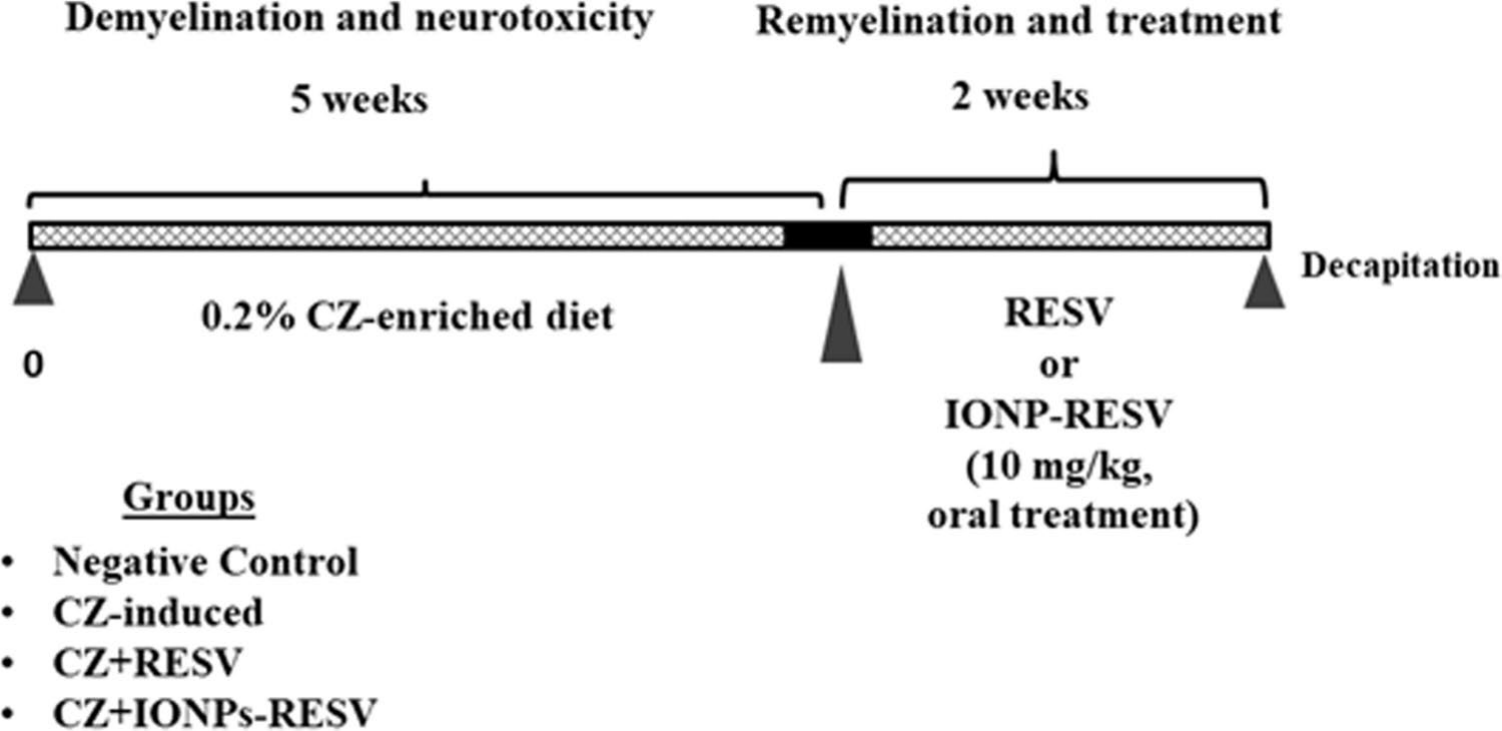
Schematic diagram illustrating the experimental duration (demyelination and treatment) and doses of RESV and IONPs-REV administrated to CZ-induced neurotoxic rats

Four groups (*n* = 10) of rats were considered, as illustrated in Fig. 1:

- Group 1: Negative control rats received a normal pelletized diet for the whole experimental period and received an oral administration of PBS 0.5 ml PBS, in the last 2 weeks.
- Group 2: CZ-neurotoxicated rats received a 0.2% CZ-enriched diet for 5 weeks.
- Group 3: CZ-RESV-treated rats received a combination of a 0.2% CZ-enriched diet for 5 weeks and an oral dose of 0.5 ml RESV (10 mg/kg) [40] for 2 weeks, starting from the 6th week after CZ intoxication.
- Group 4: CZ-IONPs-RESV-treated rats received a combination of a 0.2% CZ-enriched diet for 5 weeks and an oral dose of 0.5 ml of IONPs-RESV (10 mg/kg) for 2 weeks, starting from the 6th week after CZ intoxication. The IONPs dose (100 mg/kg) was estimated in our previous study [37].

### Tissue Collection and Processing

At the end, rats were anesthetized and subjected to a rapid cervical dislocation. The brains were excised and washed with a 0.9% sodium chloride solution. One portion of the brain “the cerebral cortex” samples was homogenized in PBS (pH 7.4) and then centrifuged at 3000 rpm for 15 min at 4 °C. The supernatants were separated, kept at − 20 °C, and used for biochemical analysis. The other portion of the brain the cerebral cortex was used for histological examination. Samples of brain tissues were immediately placed in Eppendorf tubes in liquid nitrogen at − 80 °C and used for molecular analysis.

### Biochemical Assays

#### Determination of Brain Lipid Peroxidation

Lipid peroxidation in the brain was assessed spectrophotometrically according to Ohkawa [41] by estimating malondialdehyde (MDA) content (nmol/g tissue). In which, the thiobarbituric acid reactive substance (TBARS) reacts with thiobarbituric acid to yield a pink-colored complex having absorbance at 534 nm.

#### Determination of Reduced Glutathione (GSH)

Brain GSH content (mg/g tissue) was estimated spectrophotometrically at 405 nm following Beutler [42], using a Commercial Biochemical Assay Kit (Biodiagnostic Co., Egypt). This assay is based on the reduction of DTNB by GSH to produce a yellow compound. Equal volumes of homogenate and trichloroacetic acid (500 mmol/l) were mixed and centrifuged at 3000 rpm for 15 min to separate 0.5 ml of supernatant and added to 1 ml of buffer (100 mmol/l) and 100 µl of DTNB reagent (1.0 mmol/l).

#### Determination of Brain Catalase (CAT) Activity

Brain CAT activity (units/g tissue) was assayed colorimetrically, at 510 nm according to Aebi [43], using a Commercial Biochemical Assay Kit (Biodiagnostic Co., Egypt), where 0.5 ml of 100 mM/l phosphate buffer with pH 7 was added to 100 µl of 500 mM/l H_2_O_2_ (diluted 1000 times). The reaction was started with the addition of 50 µl of the homogenate, along with subsequent addition of 200 µl of the chromogen and 500 µl of peroxidase. The decrease in the absorbance value by the hydrolysis of H_2_O_2_ of the mixture was measured with 1 min interval, after an incubation period of 10 min at 37 °C.

#### Determination of Glutathione Peroxidase (GPx) Activity

Brain GPx activity (units/g tissue) was measured spectrophotometrically, as by Paglia and Valentine [44], at 340 nm, using a Commercial Biochemical Assay Kit (Biodiagnostic Co., Egypt). In the presence of glutathione reductase (GR) and nicotinamide adenine dinucleotide phosphate (NADPH), the oxidized glutathione (GSSG) is immediately converted to the reduced form with a concomitant oxidation of NADPH to NADP. The decrease in absorbance at 340 nm is measured at 37 °C.

### Molecular Analysis of Myelin Proteolipid Protein (PLP), S100β Calcium Binding Protein (S100β), and Tumor Necrosis Factor-α (TNF-α) Genes

In order to determine the expression of genes of interest, total RNA was isolated from brain tissues by the standard TRIzol® Reagent (Invitrogen, Germany), as described by the manufacturer. RNA was reverse-transcribed into complementary DNA (cDNA) using the Arktik Thermal Cycler (Thermo Fisher Scientific Inc., USA). To evaluate the mRNA expression, qRT-PCR was conducted using QuantiTect SYBR green RT-PCR Master Mix (Qiagen, USA), as described by the manufacturer. The sequences of used primers for myelin proteolipid protein (PLP), S100β calcium binding protein (S100β), and tumor necrosis factor-α (TNF-α) are listed in Table 1. The internal reference gene “*β*-actin” was used for standardization of the analysis. Briefly, in a 25 µl reaction volume, 5 µl of cDNA was added to 12.5 µl of 2 × SYBR Green Master Mix and 200 ng of each primer. The reaction program was allocated to three steps: “The first step was at 95 °C for 3 min. The second step consisted of 40 cycles, in which each cycle was divided into three steps: (a) at 95 °C for 15 s, (b) at 55 °C for 30 s, and (c) at 72 °C for 30 s. The third step consisted of 71 cycles, which started at 60 °C and then increased by about 0.5 °C every 10 s up to 95 °C.” For data analysis, the expression of mRNAs was analyzed by the 2^−ΔΔCt^ method.

**Table 1:** The sequences of primers used in the genetic expression analysis

Gene name	Primer sequence
PLP	5′-TGGCGACTACAAGACCACCA-3′ 5′-GACACACCCGCTCCAAAGAA-3′
S100β	5′-TTGCCCTCATTGATGTCTTCCA-3′ 5′-TCTGCCTTGATTCTTACAGGTGAC-3′
TNF-α	5′-CAGGGGCCACCACGCTCTTC-3′ 5′-CTTGGGGCAGGGGCTCTTGAC-3′
*β*-Actin	5′-ACCCACACTGTGCCCATCTA-3′ 5′-CGGAACCGCTCATTGCC-3′

All primers were purchased from Qiagen (Germany)

*PLP* proteolipid protein, *S100-β* S100-β calcium binding protein, *TNF-α* tumor necrosis factor alpha

### Histopathological Examination of the Cerebral Cortex Region

Brain (cerebral cortex) tissue samples (*n* = 3 per each group) were fixed in 10% neutral buffered formalin for 72 h, then processed in serial grades of alcohol, cleared in xylene, infiltrated, and embedded into Paraplast tissue embedding media. Then, 5-µm thick sagittal sections were cut by a rotatory microtome for demonstration of the whole cerebral cortex and mounted on glass slides. Tissue sections were stained by hematoxylin and eosin (H&E) as a general morphological examination staining method, then examined by the Leica Application module attached to the full HD microscopic imaging system (Leica Microsystems GmbH, Germany). All standard procedures were done according to Culling [45].

### Statistical Analysis

The generated data were presented as mean ± standard error of the mean (SEM), *n* = 6. Statistical analyses were conducted using Statistical Package for Social Sciences (SPSS) software version 24. Simple one-way analysis of variance (ANOVA) followed by Duncan’s as post-hoc test. The difference between groups was considered significant at *P*-value < 0.05.

## Results

### FTIR Analysis

**Fig. 2:**
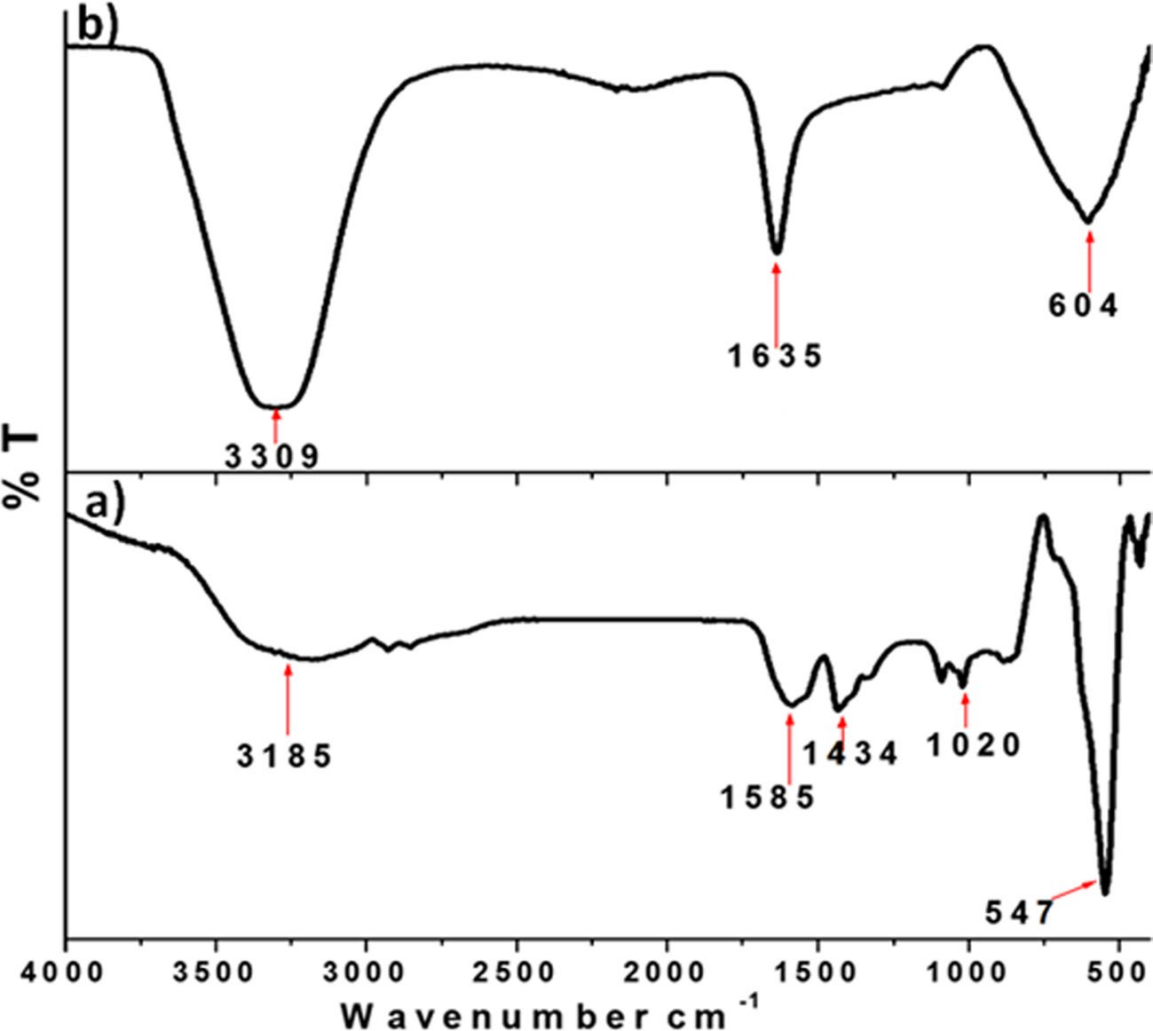
FTIR spectra of **a** iron oxide nanoparticles (IONPs) and **b** resveratrol-loaded iron oxide nanoparticles (IONPs-RESV)

FTIR spectrum of the prepared IONPs is shown in Fig. 2a. The bands that were observed at 3185 and 1585 cm^−1^ correspond to O–H absorption bands; these bands are characteristic to the absorbed water from the surrounding atmosphere [46]. According to the IR spectrum chart, the weak bands at 1020 and 1434 cm^−1^ attributed to C-O stretching of alcoholic derivatives and bending C-H, respectively, that may attribute to the presence of traces of ethylene glycol as a precursor. The strong sharp band at 547 cm^−1^ corresponds to Fe–O bond vibration modes, which is the characteristic absorption peak of magnetite Fe_3_O_4_ [47, 48]. Moreover, Fig. 2b showed the bands of IONPs-RESV which ensure the incorporation of RESV through its characteristic groups, a stretching vibration band at 3309 cm^−1^ that is attributed to OH groups and the vibration band of C = C bond in the aromatic structure is observed at 1635 cm^−1^. Furthermore, the bending vibration band of C-H in aromatic rings (δ C-H) is observed at 604 cm^−1^ [49].

### TEM Analysis of IONPs and IONPs-RESV

**Fig. 3:**
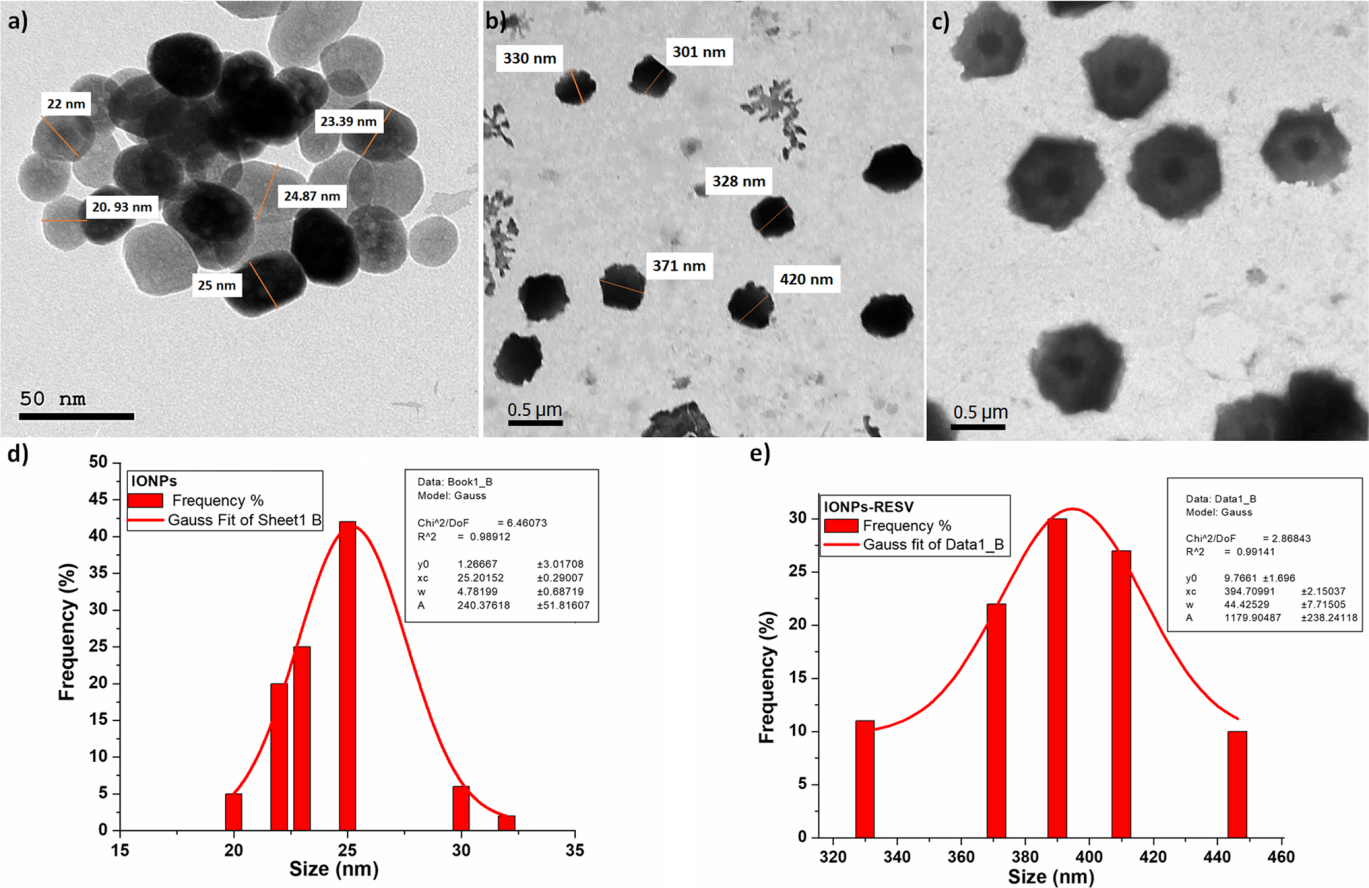
TEM images of **a** iron oxide nanoparticles (IONPs), **b** resveratrol-loaded iron oxide nanoparticles (IONPs-RESV). **c** Illustration of the shape of resveratrol-loaded iron oxide nanoparticles (IONPs-RESV). **d** Histogram of particle size distribution of iron oxide nanoparticles (IONPs). **e** Histogram of particle size distribution of resveratrol-loaded iron oxide nanoparticles (IONPs-RESV)

The size and morphology of the prepared IONPs and IONPs-RESV were evaluated by the TEM technique (Fig. 3). The TEM image (Fig. 3a) confirmed the nanosize of IONPs with the shape of nanosphere particles, and the histogram that illustrates the particle size distribution in the range of 21–25 nm was shown in Fig. 3d. Figure 3b IONPs-RESV shows a significant variation in size that changed into 301–420 nm, which was confirmed by the histogram of the particle size distribution (Fig. 3e). It is obvious from Fig. 3c that there are two regions with different brightness, where the core region (the darker) is attributed to the IONPs while the outer region (the brighter) is attributed to RESV [49–51].

### Particle Size Distribution and Zeta Potential

**Fig. 4:**
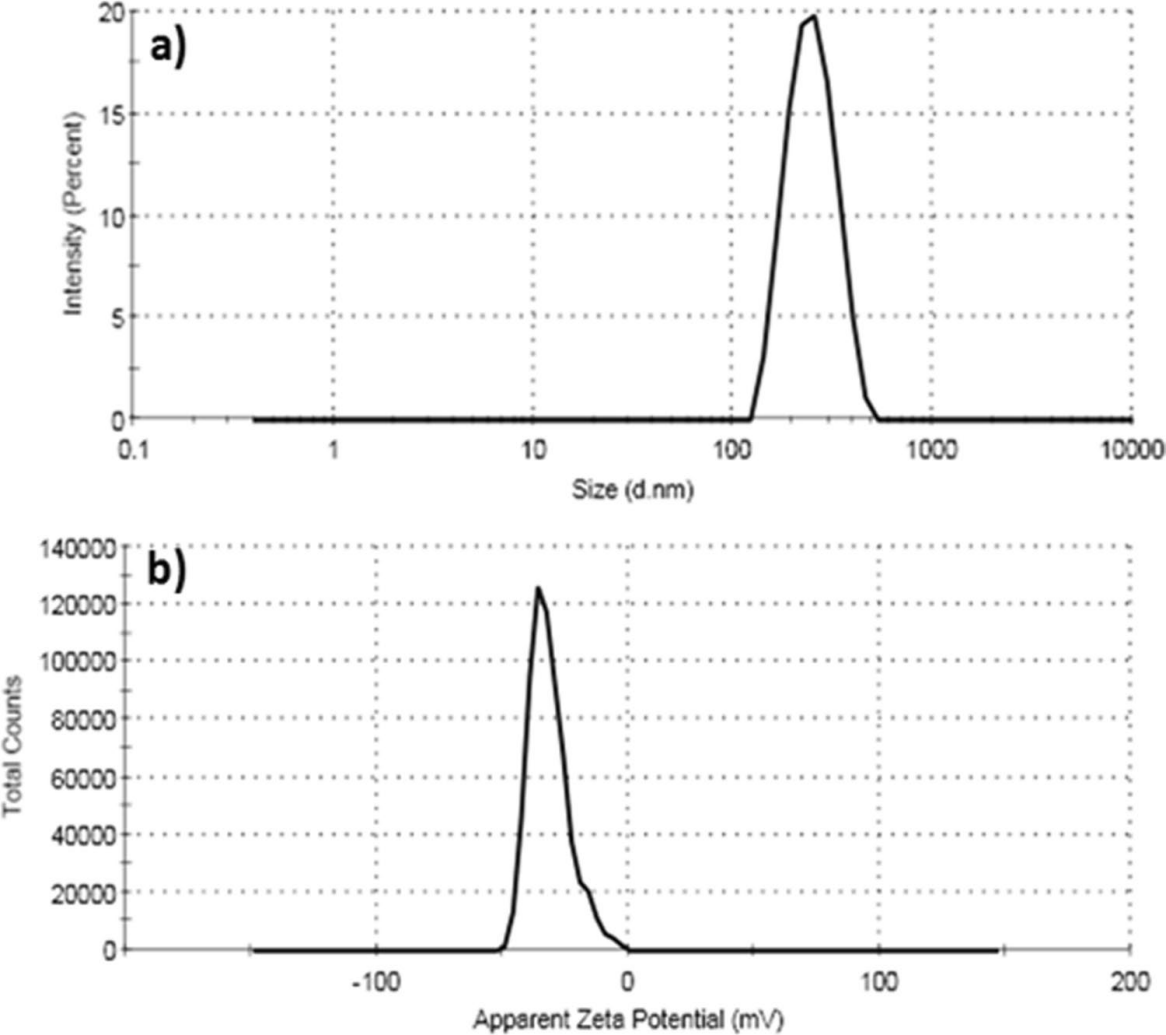
**a** Particle size distribution curve of the resveratrol-loaded iron oxide nanoparticles (IONPs-RESV) and **b** zeta potential profile of the resveratrol-loaded iron oxide nanoparticles (IONPs-RESV) recorded by Zetasizer device

The surface modification of IONPs by loading RESV is revealed by measuring the charge of IONPs-RESV using the Zetasizer instrument, where the interaction between the biomaterial and the biological cells depends on the charge of the prepared biomaterial. Figure 4a shows the particle size distribution of IONPs-RESV, which has exhibited an average dynamic distribution of particle diameters of 232.3 nm with 100% intensity. The zeta potential (Fig. 4b) of IONPs-RESV has a maximum zeta potential value at − 35.2 mV.

### X-Ray Photoelectron Spectroscopy (XPS)

**Fig. 5:**
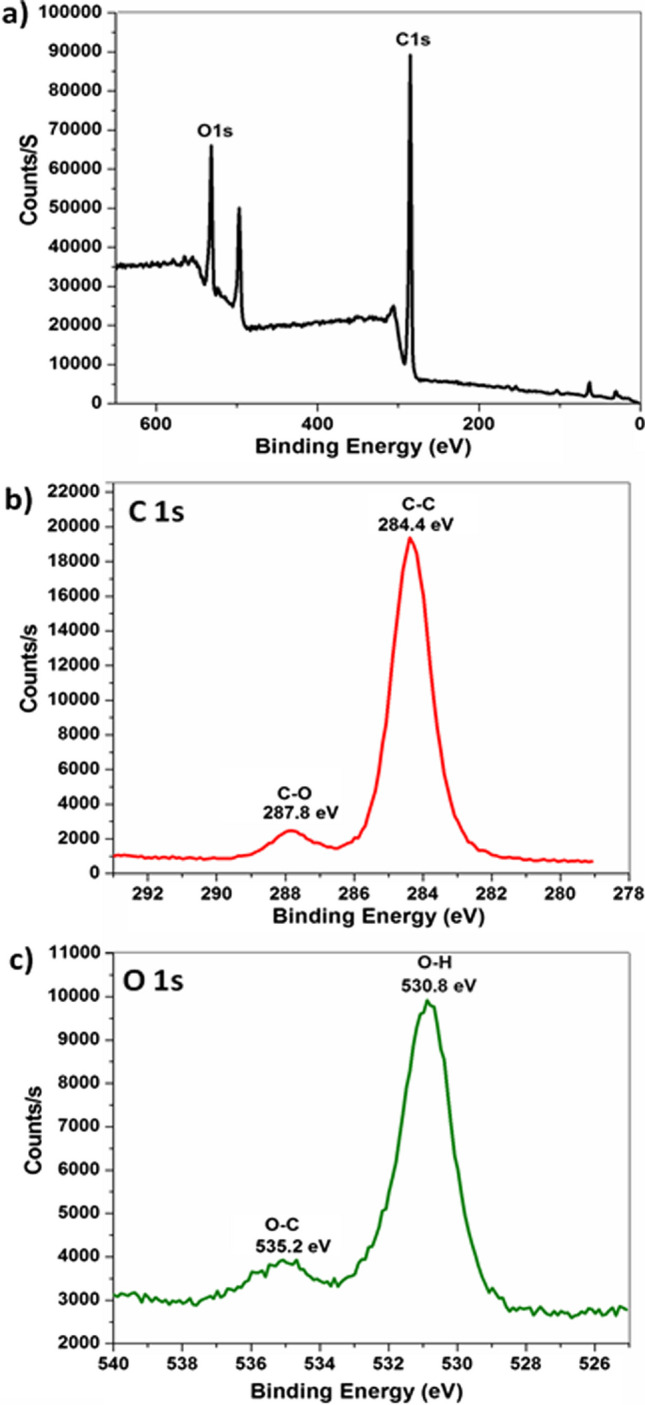
**a** XPS survey spectra of resveratrol-loaded iron oxide nanoparticles (IONPs-RESV). **b** High-resolution C 1 s. **c** High-resolution O 1 s

The technique that is used to identify the elements quantitatively that exists on the surface of a material or within it is the XPS technique. In addition, it can identify the chemical group and the chemical state (oxidation number). Figure 5 shows the XPS survey spectra of IONPs-RESV (Fig. 5a) and the high-resolution C 1 s, O 1 s XPS spectra. Based on the binding energies, the elements and the chemical functional groups could be confirmed. The elements that exist in RESV were confirmed by the XPS technique. Figure 5b shows two binding energies for C 1 s spectrum at 284.4 and 287.8 eV that correspond to C–C and C-O, respectively. The O exists at two binding energies at 530.8 and 535.2 eV in Fig. 5c for the O 1 s spectrum; these are attributed to O–H and O-C, respectively [49].

### *In Vitro* Drug Release

**Fig. 6:**
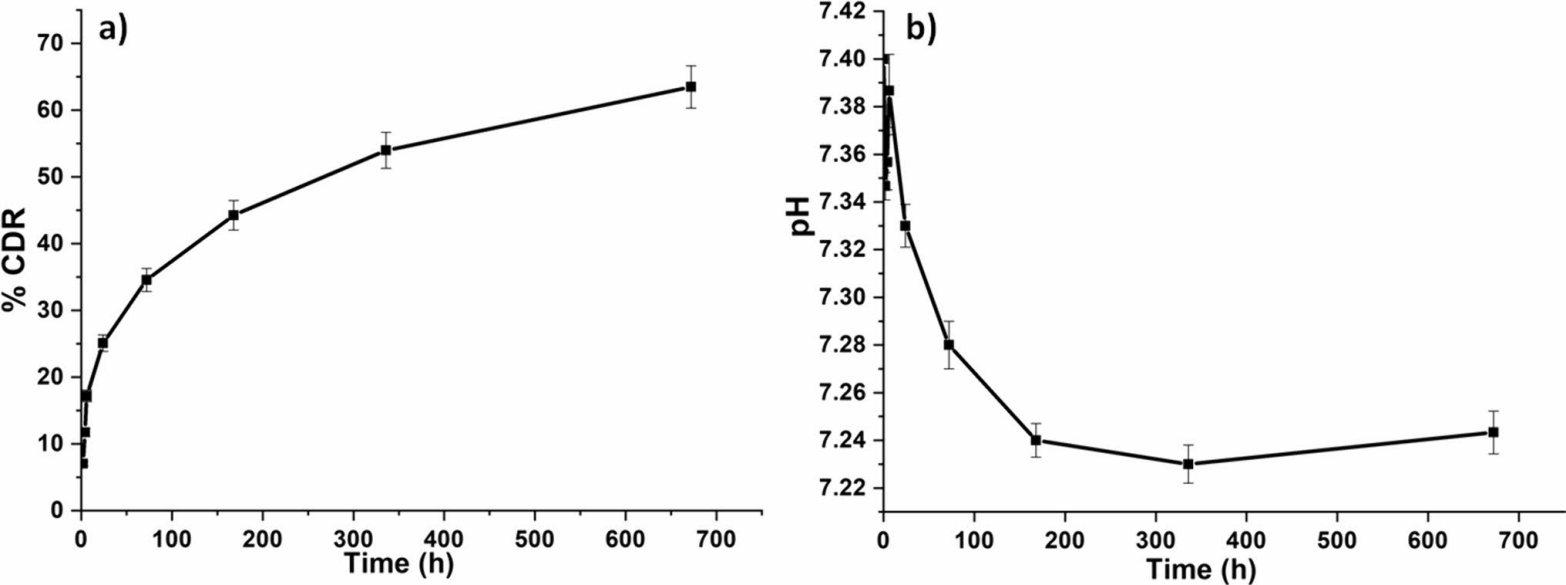
**a** Cumulative drug release (%) of resveratrol (RESV) from resveratrol-loaded iron oxide nanoparticles (IONPs-RESV) and **b** pH of PBS at time intervals (2, 4, 6, 24, 72, 168, 336, and 672 h)

The cumulative (%) drug release profiles of RESV from IONPs-RESV are represented in Fig. 6a. It is noticed that RESV is released in regular form during the studied time intervals. After 28 h, IONPs-RESV still has approximately 35% of the drug that has not been released. These results suggested that the presence of the inorganic nanomaterial (IONPs) has a positive effect on the RESV release, which resulted in a sustained release. Figure 6b shows the pH value variation of PBS solution that was measured in the mentioned time intervals (2, 4, 6, 24, 72, 168, 336, and 672 h) that started at 7.4. It was revealed that the decrease in pH value is accompanied by the increase of the release of RESV, and this may come back to its nature as a weak acid consisting of two phenols joined together by a double bond [52].

### Effect of Free RESV and IONPs-RESV on Oxidative Profile in CZ-Neurotoxicated Rats

As compared to negative control diet-fed rats, CZ-fed rats exhibited a significant reduction in brain GSH content, GPX, and CAT activities by 70, 50.2, and 49.4%, respectively. Conversely, CZ rats exhibited a significant elevation in brain MDA content (136%).

**Fig. 7:**
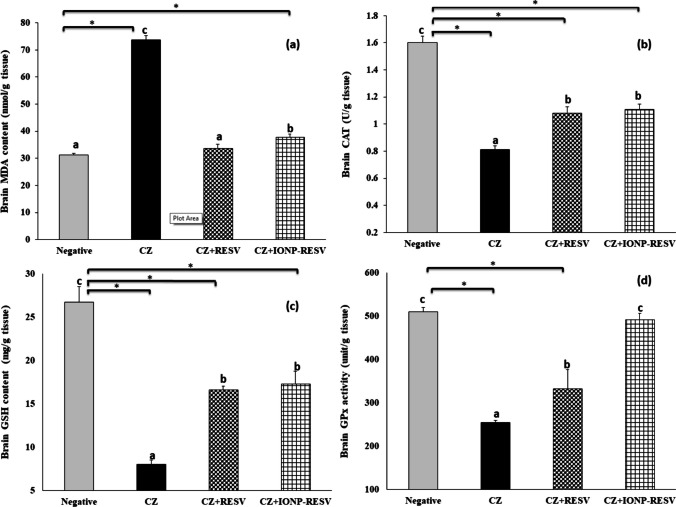
Effect of treatment of cuprizone (CZ)-induced rats with either free resveratrol (RESV) or resveratrol-loaded IONPs (IONPs-RESV) on **a** brain malondialdehyde (MDA), **b** catalase (CAT), **c** glutathione reduced (GSH), and **d** glutathione peroxidase (GPx). Data are shown as mean ± standard error of the mean (SEM), *n* = 6. Mean with different letters (a–c) is significant at *P* ≤ 0.05. Groups having the same letter are not significantly different, while those having different letters are significantly different from each other

On the other side, treatment of CZ rats with either free RESV or IONPs-RESV enhanced a significant elevation in brain GSH content (108.3 and 116.6%, respectively), brain enzymatic activities of GPX (30.8 and 93.6%, respectively), and CAT (33.5 and 36.5%, respectively), whereas MDA was significantly decreased (54.3 and 48.7%, respectively), compared to CZ-exposed brains, demonstrating the anti-oxidative potential of either free RESV or IONPs-RESV, as depicted in Fig. 7a–d.

### Effect of Free RESV or IONPs-RESV on Genetic Expression of PLP, S100β, and TNF-α

The mRNA levels of PLP, S100β, and TNF-α were measured in the brain; myelination was evaluated by PLP expression, while neuroinflammation was estimated by the genetic expression of TNF-α and S100β. As compared to control brains, CZ-fed rats showed significant downregulation of PLP (70%), along with significant upregulation of S100β and TNF-α (356 and 475%, respectively).

**Fig. 8:**
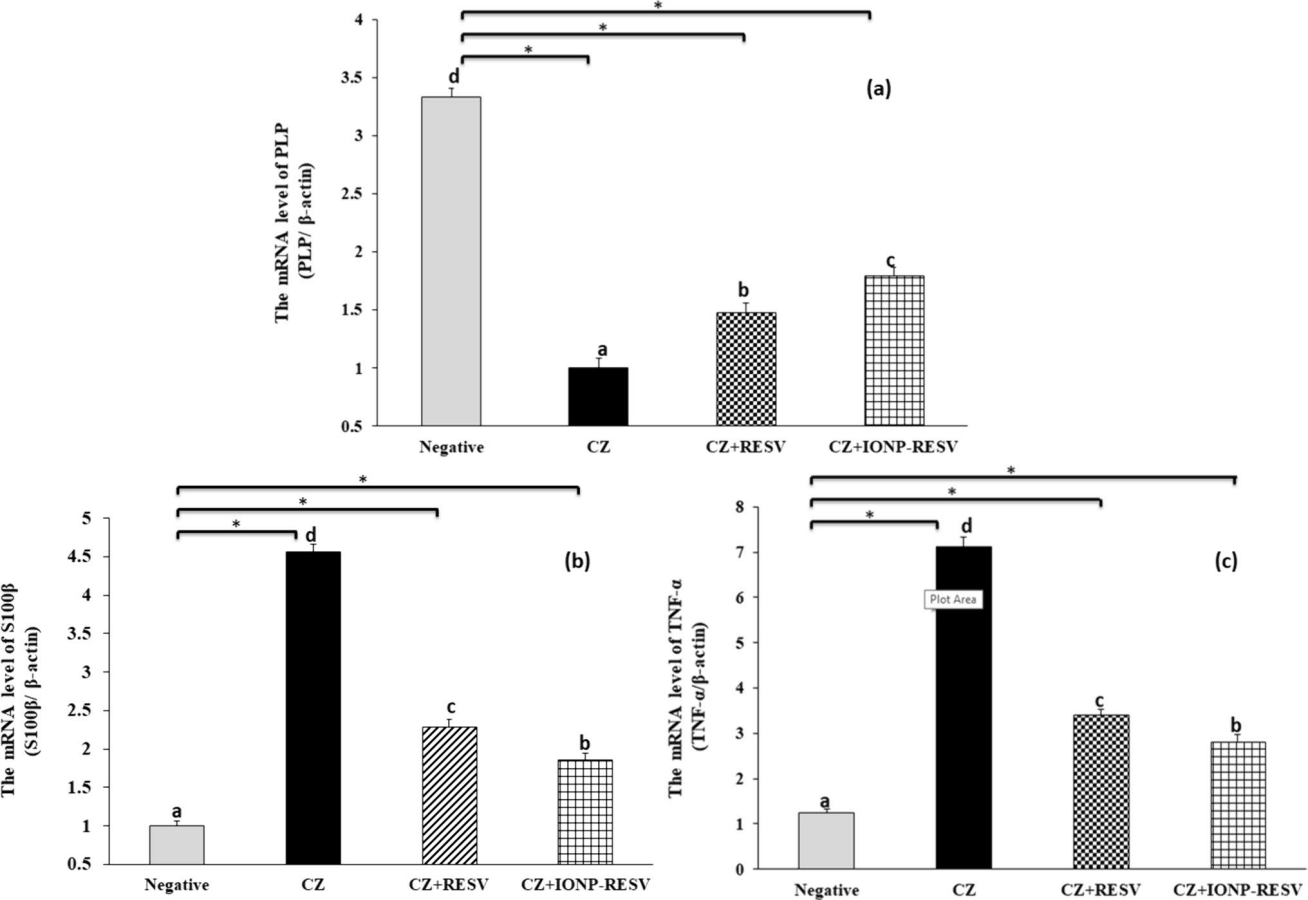
Effect of treatment of cuprizone (CZ)-induced rats with either free resveratrol (RESV) or resveratrol-loaded IONPs (IONPs-RESV) on the relative genetic expression of **a** myelin proteolipid protein (PLP), **b** S100β calcium binding protein (S100β), and **c** tumor necrosis factor (TNF-α). Data are shown as mean ± standard error of the mean (SEM), *n* = 6. Mean with different letters (a–d) is significant at *P* ≤ 0.05. Groups having the same letter are not significantly different, while those having different letters are significantly different from each other

As compared to CZ-induced brains, treatment with either RESV or IONPs-RESV resulted in a significant upregulation in PLP (48.5 and 80.3%, respectively), as well as a significant downregulation of S100β (49.7 and 59.2%, respectively) and TNF-α (52.2 and 60.5%, respectively). Both RESV and IONPs-RESV amended these CZ-induced alterations in myelination as indicated by a marked upregulation of PLP expression and significant downregulation in S100β and TNF-α, demonstrating the remyelination potential and anti-inflammatory activity. More interestingly, those MS-related genes’ mRNA levels in treated CZ-brains with IONPs-RESV were significantly higher downregulated than those that were treated with free RESV (Fig. 8a–c). Table 2 represents the analysis of the abovementioned data.

**Table 2:** Statistical report for ANOVA, *F*, df, and exact *P*-values

		Sum of squares	df	Mean square	*F*	Sig
MDA	Between groups	6978.610	3	2326.203	278.693	0.000
	Within groups	150.243	18	8.347		
	Total	7128.853	21			
CAT	Between groups	1.509	3	0.503	87.461	0.000
	Within groups	0.069	12	0.006		
	Total	1.578	15			
GSH	Between groups	954.721	3	318.240	47.062	0.000
	Within groups	101.431	15	6.762		
	Total	1056.153	18			
GPx	Between groups	237,237.865	3	79079.288	41.350	0.000
	Within groups	28,686.616	15	1912.441		
	Total	265,924.481	18			

### Effect of RESV or IONPs-RESV on CZ-Induced Histopathological Alterations in the Cerebral Cortex

**Fig. 9:**
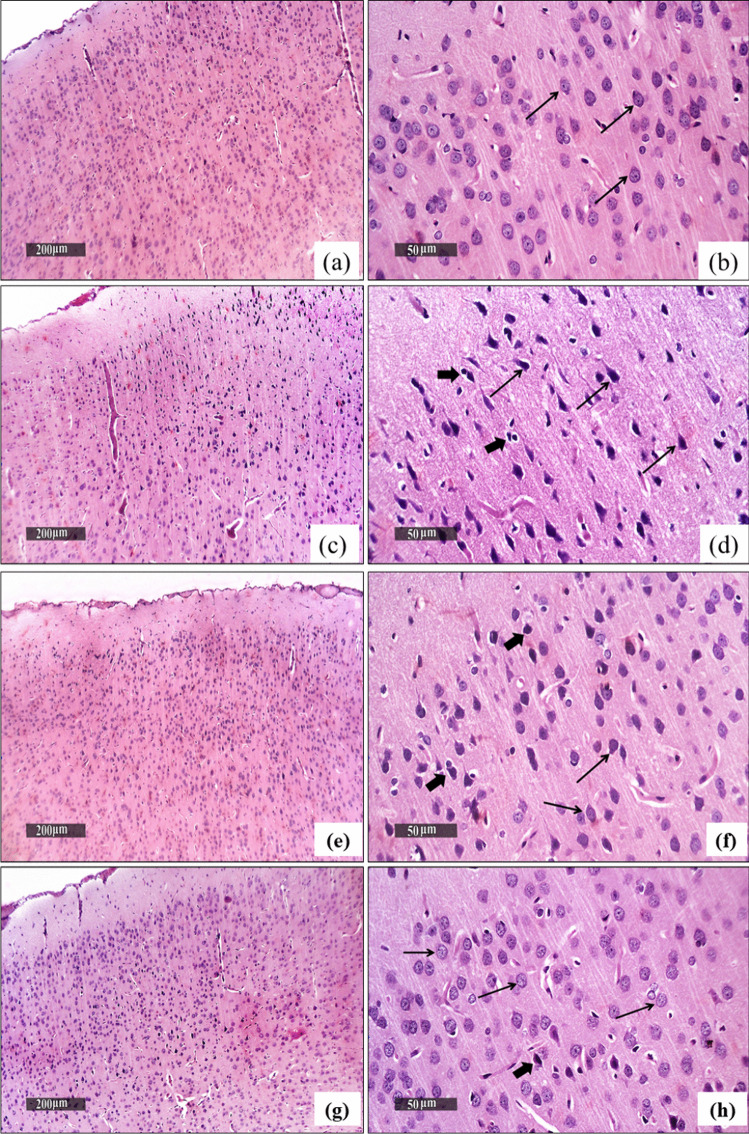
Effect of treatment of cuprizone (CZ)-induced rats with either free resveratrol (RESV) or resveratrol loaded on IONPs (IONPs-RESV) on histopathological changes in the brain tissues of different groups: **a**, **b** Negative control brains showing normal morphological features with intact intercellular brain matrix and well-organized neurons with intact subcellular details (thin arrow). **c**, **d** CZ-brains showing wide areas of diffuse neuronal damage and degenerative alterations in the cortical layers with several figures of shrunken, darkly stained, and pyknotic neurons (thin black arrow), accompanied with mild perineuronal edema and vacuolization of brain matrix with mild higher reactive glial cells infiltrates (thick black arrow). **e**, **f** CZ-treated rats with free RESV showed almost intact brain matrix with few focal neuronal degenerative changes in outer cortical layers (thick black arrow), with many records of apparent intact neurons (thin black arrow), and almost intact brain matrix. **g**, **h** CZ-treated rats with IONPs-RESV showed almost well-organized morphological features including intact cortical neurons (thin black arrow), with minimal scattered records of neuronal degenerative changes, with minimal glial cells infiltrates (thick black arrow)

Negative control brains exhibited normal morphological properties of cerebral cortex layers, with well-organized neurons with intact subcellular details, and showed an intact intercellular brain matrix with normally distributed glial cells with intact vasculatures (thin arrow) (Fig. 9a, b). On the other side, CZ-neurotoxicated rats showed wide areas of diffuse neuronal damage and degenerative alterations in outer and inner cortical layers with several figures of shrunken, darkly stained, and pyknotic neurons (thin black arrow), associated with mild perineuronal edema and vacuolization of the brain matrix, with mildly higher reactive glial cells infiltrates (thick black arrow) (Fig. 9c, d). Conversely, treatment of CZ rats with free RESV enhanced a significant neuroprotective impact, as evidenced by the few focal neuronal degenerative changes in the outer cortical layers (thick black arrow) with many records of apparent intact neurons (thin black arrow) and an almost intact brain matrix (Fig. 9e, f). More interestingly, treatment of CZ rats with IONPs-RESV (Fig. 9g, h) showed higher neuroprotective potential with almost well-organized morphological features, including intact cortical neurons (thin black arrow) with minimal scattered records of neuronal degenerative changes, along with an intact brain matrix with minimal glial cells infiltrates (thick black arrow).

## Discussion

In this research study, we have demonstrated that administration of either free RESV or IONPs-RESV at 10 mg/kg for 2 weeks to CZ-neurotoxicated rats significantly exhibited neurotherapeutic potential manifested as anti-oxidative, anti-inflammatory, and remyelination activities. Also, IONPs-RESV was more potent than free RESV against CZ-induced MS and neurotoxicity in the cortical region. The loading of RESV onto the Fe_3_O_4_ nanoparticles (IONPs) was assured by the FTIR, TEM, zeta potential, and XPS techniques. These characterizations revealed that the physical and chemical features of RESV were maintained after loading onto the IONPs. The sustained release behavior of RESV was recognized from the obtained nanoformulations; up to 65% of the drug was released after 28 days of in vitro release. It is worthy to highlight that the sustained drug release profiles are favored for the treatment of neural disorders, along with decreasing the possible side effects [53–55]. Moreover, this study demonstrated that IONPs loaded with resveratrol (IONP-RESV) had more potent anti-inflammatory effects in a rat model of demyelination induced by CZ, in comparison to free RESV. They exhibited more efficacy than free RESV in counteracting the decrease in myelin PLP production caused by CZ, as well as the upregulation of the inflammatory markers TNF-α and S100β. The histopathological analysis of the cerebral cortex indicated that IONP-RESV provided superior neuroprotective advantages compared to free RESV in alleviating demyelination and neuroinflammation. However, the utilization of a particular CZ-induced demyelination model, which may not comprehensively depict the intricate pathophysiology of MS, necessitates additional thorough investigations and clinical trials to authenticate the findings.

Our findings demonstrated that CZ administration to rats enhanced a significant lipid peroxidation coupled with a significant decline in the anti-oxidative activities of GSH, GPx, and CAT in the brain. This might be ascribed to the vulnerability of the brain to oxidative insult due to its relatively high oxygen consumption, elevated content of polyunsaturated fat, and decreased antioxidant levels [56, 57]. This CZ-provoked oxidative stress is in parallel with the previous studies [1, 31, 58]. MDA is “a neurotoxic and pro-inflammatory compound,” which could link oxidative activities with demyelination [59]. Moreover, CZ-induced histopathological alterations could be attributed to the copper-chelating nature of CZ which in turn resulted in exacerbated oxidative stress and stimulated lipid peroxidation that resulted in the degradation of cellular membranes and neuroinflammation [39]. The great abundance of lipids “about 70%” of myelin dry mass renders lipid peroxidation highly involved in CZ-induced demyelination; it was found that MS patients exhibited a higher degree of lipid peroxidation in the cerebrospinal fluid (CSF) [60].

Treatment of CZ rats with RESV and IONPs-RESV demonstrated anti-oxidative potential, restored the antioxidant parameters (CAT, GPx, and GSH), and decreased brain MDA contents; therefore, RESV and IONPs-RESV conferred protection of neuronal function and integrity from oxidative insults. Antioxidant activities of “GPx and CAT” are the first line of defense against oxidative insults [39]. Both GPx and CAT functions to get rid of the additional hydroperoxides and to detoxify H_2_O_2_ to H_2_O and thus contributing to the antioxidant protection in the neural tissues [61]. This anti-oxidative potential is ascribed to the nature of RESV, as an excellent antioxidant, due to its ability to suppress lipid peroxidation and to enhance the upregulation of some antioxidant enzymes in the brain [62]. In addition, trans-RESV is the stereoisomer that was discovered to exhibit a multitude of health-promoting benefits; RESV demonstrated potentials in the areas of anti-oxidative and anti-inflammatory effects [14–16]. In addition, RESV was shown to exhibit neuroprotective properties against a number of neurological illnesses [11–13]. In individuals suffering from Alzheimer’s disease, RESV was found to have neurotherapeutic, anti-inflammatory, and immunomodulatory effects, according to the findings of a recent clinical trial (NCT01504854) [17].

In addition, this resemblance of the anti-oxidative potential of both RESV formulation, except for MDA and GPx, indicated that nanoparticles did not stimulate ROS generation-associated nanotoxicity and thus exhibit a safe toxicological profile. Ashafaq et al. have evaluated the anti-oxidative activity of RESV and its nanoformulation indicated that nano-RESV (125, 250, and 500 µg) has 40–800 times more efficacy than free RESV [22]. The nanoformulation of this natural ROS scavenger renders it more potent at a low molecular size, through facilitating the penetration of neurons and subsequent protection from oxidative stress. Herein, IONPs-RESV stimulated GPx activity higher than RESV.

Furthermore, our findings showed that CZ intoxication promoted the downregulation of the mRNA levels of the myelin-related gene “PLP” along with the upregulation of the mRNA levels of S100β. MS is characterized by the loss of myelin sheath; PLP is one of the main myelin proteins that results in the “multilayered structure” of myelin [63]. Regarding PLP, our findings are in agreement with previous studies [31] and [64], which found PLP expression was significantly decreased after five weeks of CZ intoxication.

On the other hand, CZ administration enhanced the upregulation of S100β gene expression. Elevated levels of inflammatory S100β might enhance glial reactivity and oligodendrocyte disruption, enhancing neurodegeneration or delaying the following remyelination [65]. Intracellularly, S100β functions as a signaling molecule that is capable of promoting neuronal proliferation, oligodendrocyte differentiation, and facilitating astrocyte and microglia migration [66]. At low concentrations, S100β could enhance neuronal survival and stimulate astrocytic proliferation [67]. On the other hand, S100β reaches micromolar concentrations and exerts neurotoxic effects under stress conditions through stimulating the release of inflammatory and oxidative stress mediators and enhancing microglial and astrocyte activation [68]. This finding runs in parallel with our previous study [31]; therefore, downregulation of S100β in the current treated CZ-induced model signifies the importance of S100β as a diagnostic biomarker and as an emerging therapeutic target in MS.

Moreover, CZ brains demonstrated increased mRNA levels of TNF-α; our findings are in agreement with Voß [69] and Ibrahim and Ahmed [70], while in contrast with Raasch [71] and [58], who demonstrated that CZ-intoxicated rats exhibited an insignificant increase in TNF-α level. Brain injury stimulates the release of TNF-α, which subsequently enhances the propagation of activated microglia and intensifies further neuroinflammation [22]. It was observed that the early microglial activation in demyelination is followed by elevated expression of inflammatory mediators [72], including TNF-α and S100β, as demonstrated in this study.

Upon treatment of CZ rats with both RESV and IONPs-RESV, the increased expression of TNF-α was significantly declined. This is in agreement with previous studies [73, 74] that evidenced the neuroprotective potency of RESV through its ability to balance the pathways of pro-inflammatory and anti-inflammatory cytokines; RESV and IONPs-RESV maintain BBB integrity in the treated CZ-intoxicated rats through inhibiting microglial activation.

Treatment of CZ rats with RESV and IONPs-RESV significantly upregulated PLP expression (48.5 and 80.3%, respectively) and downregulated S100β expression (49.7 and 59.2%, respectively) and TNF-α expression (52.2 and 60.5%, respectively); these results demonstrated the higher potential of loaded RESV within IONPs to halt demyelination, to initiate remyelination, and to suppress neuroinflammation. This might be ascribed to the limited efficacy of polyphenol-based treatment, because of their low solubility, limited bioavailability, and rapid degradation after intestinal absorption and enterohepatic metabolism. Therefore, nanoformulation of polyphenols can improve their solubility and stability, increase their absorption, protect them from pre-mature in vivo degradation in the body, and extend their circulation time [75, 76]. The administration of different RESV-loaded nanocarriers extends plasma RESV concentrations for longer periods and ameliorates other pharmacokinetic parameters, due to the nanocarriers’ resistance to hepatic metabolism [77].

These molecular and biochemical results were supported by the histological findings of the cerebral cortices of different groups that illustrated the neurodegenerative potential of CZ and vacuolization of the brain matrix, with mild higher reactive glial cells infiltrates, as well as the neuroprotective action with intact cortical neurons of either RESV or IONPs-RESV. Histopathologically, IONPs-RESV demonstrated higher neuroprotective potential.

Collectively, our findings demonstrated that the neuroprotective potential of IONPs-RESV is better than that of free RESV; this might be explained by the ability of IONPs to improve RESV’s permeability into the neural cells to exert their potent anti-inflammatory activities. This confirmed that using nanocarriers enhances the in vivo stability of RESV and its pharmacological concentrations in the brain, thus strengthening its neurotherapeutic potential [78]. Accordingly, the formulated IONPs-RESV offers therapeutic potential for brain delivery to halt demyelination and initiate remyelination in a CZ-induced rat model. Finally, this study was conducted on male rats, as a previous study by Ünsal and Özcan [79] showed that both males and females are affected by CZ-induced neurotoxicity, but males are affected more than females.

## Conclusion

Nanoformulations of magnetite loaded with resveratrol (IONP-RESV) were successfully designed and characterized in this study. Up to 65% of the drug was released after 28 days *of *in vitro evaluation, indicating the potential for enhanced patient satisfaction and reduced side effects. This IONP-RESV nanoformulation exhibited greater neurotherapeutic potential against CZ-induced neurotoxicity and multiple sclerosis compared to free RESV. While these results are promising, it is important to acknowledge the limitations of the study. Notably, the assessment of neurobehavioral parameters, neuronal proliferation, oligodendrocyte differentiation, and astrocyte and microglia markers was not included, which would have provided a more comprehensive understanding of the neuroprotective mechanisms. Additionally, the extrapolation of the findings from the in vitro and animal models to human clinical applications requires caution, as the complex pathophysiology of multiple sclerosis may not be fully captured in these experimental settings. Furthermore, the fate and potential nanotoxicity of the IONPs-RESV system require further in-depth investigation. Future studies should also expand the analysis to different brain regions to elucidate the regional-specific effects of the nanoformulation. Overall, the RESV-loaded IONPs represent a promising approach for the treatment of neurodegenerative disorders, such as demyelinating conditions. However, to fully harness the potential of this technology, larger and more randomized clinical trials are necessary to estimate the real contribution of nanotechnology in the delivery of RESV for the management of brain disorders.

## Data Availability

The data and materials are available upon request.

## Abbreviations

ANOVA: One-way analysis of variance
BBB: Blood-brain barrier
BCS: Biopharmaceutics Classification System
CAT: Catalase
cDNA: Complementary DNA
CNS: Central nervous system
CSF: Cerebrospinal fluid
Cu: Copper
CZ: Cuprizone
FTIR: Fourier transformer infrared
GPx: Glutathione peroxidase
GSH: Glutathione
H&E: Hematoxylin and eosin
IONPs: Iron oxide nanoparticles
MDA: Malondialdehyde
MS: Multiple sclerosis
NPs: Nanoparticles
NRC: National Research Center
PBS: Phosphate saline buffer
PLP: Proteolipid protein
qRT-PCR: Quantitative reverse transcription–polymerase chain reaction
RESV: Resveratrol
ROS: Reactive oxygen species
S100-β: S100-β calcium binding protein
SEM: Standard error of the mean
SPSS: Statistical Package for Social Sciences
TEM: Transmission electron microscopy
TNF-α: Tumor necrosis factor-α
UHV: Ultrahigh vacuum
XPS: X-ray photoelectron spectroscopy
